# Efficacy and safety of zoledronic acid versus denosumab in the treatment of primary osteoporosis: a single-center retrospective cohort study

**DOI:** 10.3389/fendo.2026.1825856

**Published:** 2026-08-18

**Authors:** Xuan Li, Kejia Huang, Lanling Zhang, Yeqing Shi, Jinhe Zhao, Xiaofang Li, Jie Gao, Dongbao Zhao, Wei Wan

**Affiliations:** 1 Fangta Traditional Chinese Medicine Hospital of Songjiang District of Shanghai, Shanghai, China; 2 School of Basic Medical Sciences, Naval Medical University, Shanghai, China; 3 Department of Rheumatology and Immunology, First Affiliated Hospital of Naval Medical University, Shanghai, China

**Keywords:** bone mineral density, bone turnover markers, denosumab, primary osteoporosis, zoledronic acid

## Abstract

**Objective:**

To compare the long-term efficacy and safety of zoledronic acid versus denosumab in the treatment of primary osteoporosis.

**Methods:**

This retrospective cohort study analyzed 328 patients diagnosed with primary osteoporosis at the Department of Rheumatology and Immunology, Changhai Hospital, between January 2017 and December 2020. After applying inclusion and exclusion criteria, 164 patients were enrolled and divided into the zoledronic acid group (n = 88) and the denosumab group (n = 76). Bone mineral density (BMD) at the lumbar spine (L1–L4) and hip, bone turnover markers (including bone alkaline phosphatase, serum C-terminal telopeptide of type I collagen, and serum N-terminal propeptide of type I procollagen), and adverse events were evaluated at baseline and at 1, 2, and 3 years post-treatment.

**Results:**

At baseline, the two groups exhibited no statistically significant differences in most parameters except for parathyroid hormone (PTH), lumbar spine BMD, and lumbar T-score. After three years of treatment, denosumab demonstrated superior efficacy compared to zoledronic acid in key outcome measures, including lumbar spine BMD (0.85 ± 0.17 vs. 0.81 ± 0.14), hip BMD (0.85 ± 0.13 vs. 0.79 ± 0.12), and β-CTX (0.27 ± 0.19 vs. 0.38 ± 0.23), with statistically significant differences (P < 0.05). Both treatments demonstrated favorable safety profiles, with no serious adverse events reported.

**Conclusion:**

Denosumab exhibits superior long-term efficacy over zoledronic acid in improving bone mineral density and suppressing bone turnover. Both agents demonstrate excellent safety profiles and represent effective therapeutic options for the management of osteoporosis.

## 1 Introduction

Osteoporosis (OP) is a systemic skeletal disorder characterized by reduced bone mass and deterioration of bone microarchitecture, leading to increased bone fragility and elevated fracture risk (1, 2). As a major global public health concern, osteoporosis significantly impairs quality of life in the elderly population and imposes substantial socioeconomic burdens. Statistics indicate that an osteoporotic fracture occurs every three seconds worldwide, with vertebral and hip fractures being the most common types. The associated disability and mortality rates are comparable to those observed in advanced-stage malignancies (1). China has one of the largest populations of osteoporosis patients globally. With the accelerating pace of population aging, the prevention and treatment of osteoporosis have become increasingly critical priorities (3).

Primary osteoporosis (POP) represents the most common form of osteoporosis and is primarily classified into postmenopausal osteoporosis and senile osteoporosis. The pathophysiological basis involves an imbalance between bone resorption and bone formation, wherein the rate of bone resorption exceeds that of bone formation, resulting in progressive bone loss (4). Bone mineral density (BMD) serves as the gold standard for osteoporosis diagnosis and can be measured using dual-energy X-ray absorptiometry (DXA). A T-score below -2.5 standard deviations (SD) confirms the diagnosis (4). Additionally, bone turnover markers (BTMs), such as serum C-terminal telopeptide of type I collagen (β-CTX) and N-terminal propeptide of type I procollagen (P1NP), sensitively reflect dynamic changes in bone metabolism (5, 6). β-CTX is a specific marker of bone resorption, while P1NP is a specific marker of bone formation (7). These markers provide valuable insights for assessing drug efficacy and predicting fracture risk.

Currently, anti-osteoporotic medications are primarily categorized into anti-resorptive agents and anabolic agents. Zoledronic acid, a nitrogen-containing bisphosphonate, exerts its therapeutic effect by inhibiting osteoclast activity, thereby reducing bone resorption (8, 9). Its notable advantage lies in the convenient dosing schedule, requiring only a single annual intravenous infusion (10). Denosumab, a fully humanized monoclonal antibody, specifically targets the receptor activator of nuclear factor-κB ligand (RANKL), preventing the binding of RANKL to RANK receptors on osteoclast precursor cells. This mechanism inhibits osteoclast formation, function, and survival (11, 12). Denosumab demonstrates potent therapeutic efficacy and is administered via subcutaneous injection every six months (13).

Although both zoledronic acid and denosumab have demonstrated clinical efficacy and are recommended anti-resorptive therapies for osteoporosis, current guidelines do not clearly define a treatment priority between these two agents for patients with primary osteoporosis. In routine clinical practice, the choice between zoledronic acid and denosumab is often influenced by patient characteristics, expected treatment duration, adherence, safety concerns, and physician preference. Previous investigations have suggested that denosumab may be superior to zoledronic acid in increasing BMD and reducing fracture risk (14, 15). However, other studies have reported comparable efficacy between the two agents in fracture risk reduction (16, 17). Furthermore, denosumab discontinuation may result in a rebound phenomenon, characterized by elevated bone turnover markers and decreased bone mineral density, potentially increasing the risk of multiple vertebral fractures (18–20). Therefore, this retrospective study aims to compare the long-term effects of zoledronic acid and denosumab on bone mineral density, bone turnover markers, and safety profiles in patients with primary osteoporosis under real-world conditions, thereby providing clinicians with valuable evidence to guide therapeutic decision-making.

## 2 Methods

### 2.1 Study participants

This single-center, retrospective cohort study enrolled patients diagnosed with primary osteoporosis at the Department of Rheumatology and Immunology, Changhai Hospital, between 1. All data were extracted from anonymized medical records, with no patient privacy information involved. The study protocol was approved by the Ethics Committee of Changhai Hospital (Approval No. B2022-055) and conducted in accordance with the principles of the Declaration of Helsinki.

**Figure 1:**
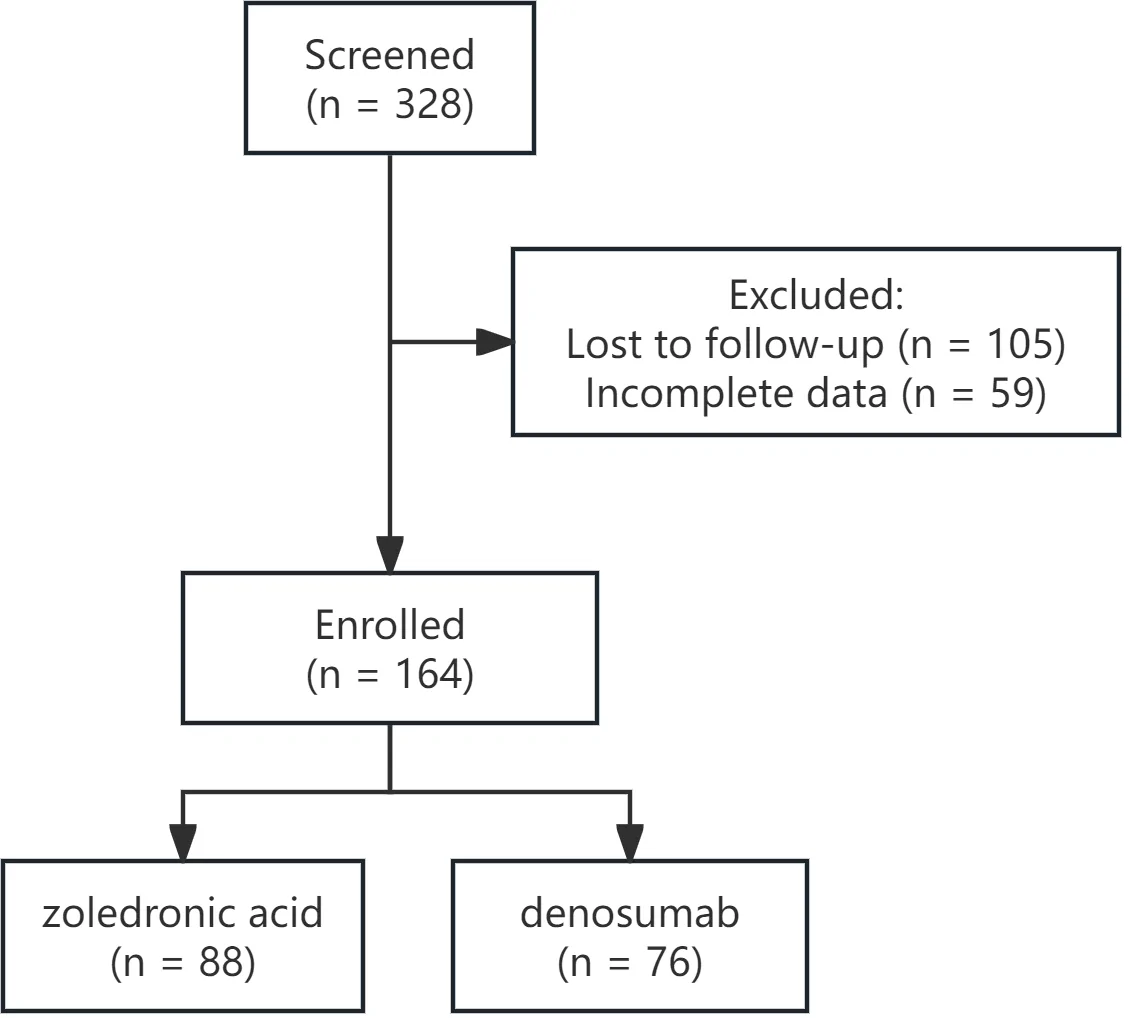
Research flow chart.

Patients diagnosed with primary osteoporosis at the Department of Rheumatology and Immunology, Changhai Hospital, between January 2017 and December 2020 were retrospectively screened through the hospital electronic medical record system. A total of 328 patients were initially identified. After excluding patients with incomplete clinical data or loss to follow-up, 164 patients were included in the final analysis, as illustrated in **Figure 1**. Participants were selected based on the following inclusion and exclusion criteria.

Inclusion Criteria:

1. Diagnosis of primary osteoporosis according to the Chinese Guidelines for the Diagnosis and Treatment of Primary Osteoporosis (2022) (21): postmenopausal women or men aged 50 years and older with T-scores ≤ -2.5 SD at the lumbar spine, femoral neck, or total hip as measured by DXA;
2. Age > 50 years;
3. Treatment with either zoledronic acid (5 mg, intravenous infusion, annually, Novartis AG, Swiss) or denosumab (60 mg, subcutaneous injection, every six months, Amgen, USA) with a minimum follow-up duration of 3 years;
4. Complete BMD and bone turnover marker data available at baseline and at 1, 2, and 3 years post-treatment.

Exclusion Criteria:

1. Secondary osteoporosis, such as that caused by endocrine disorders, rheumatic diseases, or medications (e.g., glucocorticoids);
2. Prior treatment with other anti-osteoporotic medications (e.g., bisphosphonates, teriparatide);
3. Severe hepatic or renal impairment, abnormal serum calcium levels, or parathyroid dysfunction;
4. Active infection, malignancy, or metabolic bone diseases;
5. Loss to follow-up or incomplete data.

### 2.2 Data collection and outcome measures

Clinical data at baseline and at 1, 2, and 3 years post-treatment were collected through the hospital’s electronic medical record system, including:

Demographic Information: Age and sex.

Bone Mineral Density: BMD (g/cm²) and corresponding T-scores at the lumbar spine (L1–L4) and hip (total hip or femoral neck) were measured using dual-energy X-ray absorptiometry (DXA, GE Medical Systems Lunar, USA).

Bone metabolism markers included serum β-CTX, serum P1NP, 25-hydroxyvitamin D (25-OH VitD), and parathyroid hormone (PTH), which were measured using commercially available assays from Roche Diagnostics.

Safety Assessment: All adverse events during the treatment period were recorded, including acute-phase reactions, hypocalcemia, osteonecrosis of the jaw, and atypical femoral fractures.

### 2.3 Statistical analysis

Statistical analysis was performed using SPSS version 26.0 software. Continuous variables with normal distribution were expressed as mean ± standard deviation (SD) and compared using independent samples t-tests. Non-normally distributed continuous variables were analyzed using the Mann-Whitney U test. Categorical variables were presented as percentages and compared using chi-square tests. Repeated measures analysis of variance (ANOVA) was employed to evaluate changes in outcome measures across different time points. To address potential baseline imbalance, an additional analysis of covariance (ANCOVA) was performed for hip BMD at 3 years. Treatment group was included as the fixed factor, while age, sex, baseline PTH, baseline lumbar spine BMD, baseline lumbar spine T-score, and baseline hip BMD were included as covariates. Because of the retrospective design, no prospective sample size calculation was performed. All eligible patients who met the predefined inclusion and exclusion criteria during the study period were included. *Post-hoc* power analysis was conducted using the pwr package in R software, based on the observed between-group differences in hip BMD and β-CTX at 3 years. The *post-hoc* power analysis based on the observed difference in hip BMD at 3 years showed that the current sample size provided approximately 86% power to detect the between-group difference. All statistical tests were two-sided, and P-values < 0.05 were considered statistically significant.

## 3 Results

### 3.1 Baseline characteristics

A total of 328 patients were initially screened, of whom 164 with complete data were enrolled, including 88 in the zoledronic acid group and 76 in the denosumab group. The zoledronic acid group comprised 10 males and 78 females, with a mean age of 66.13 ± 10.15 years. The denosumab group included 3 males and 73 females, with a mean age of 65.67 ± 10.18 years. No statistically significant differences were observed between the two groups in baseline parameters including age, sex, NMID, P1NP, 25OH-VitD, hip BMD, and hip T-score (P > 0.05), indicating comparable baseline characteristics. However, significant differences were noted at baseline in PTH (43.34 ± 15.21 vs. 37.26 ± 10.17), lumbar spine L1–4 BMD (0.69 ± 0.15 vs. 0.64 ± 0.12), and lumber T-score (-2.88 ± 0.59 vs. -3.12 ± 0.59) between the zoledronic acid and denosumab groups, respectively (P < 0.05).

### 3.2 Bone turnover markers

Both treatment groups demonstrated significant reductions in β-CTX levels post-treatment; however, the magnitude of reduction differed between groups. β-CTX levels in the denosumab group were significantly lower than those in the zoledronic acid group at both 2 and 3 years post-treatment (P = 0.025 and P = 0.002, respectively), indicating more potent suppression of bone resorption with denosumab.

P1NP levels decreased in both groups post-treatment; however, no statistically significant differences were observed between groups at 1, 2, or 3 years post-treatment (P > 0.05). These findings suggest that both medications effectively suppress bone turnover, with denosumab demonstrating superior efficacy in inhibiting bone resorption.

PTH levels at baseline were significantly lower in the denosumab group compared to the zoledronic acid group. Post-treatment, PTH levels decreased progressively in both groups; however, the denosumab group exhibited a greater magnitude of reduction and consistently lower PTH levels compared to the zoledronic acid group (P < 0.001), representing a highly statistically significant difference (**Table 1**).

**Table 1:** Comparison of bone turnover markers before and after treatment.

Parameter	Treatment	n	Pre-treatment	1 year	2 years	3 years
β-CTX (ng/mL)	Zoledronic Acid	88	0.55 ± 0.30	0.48 ± 0.27	0.43 ± 0.25	0.38 ± 0.23
	Denosumab	76	0.57 ± 0.29	0.45 ± 0.25	0.35 ± 0.21	0.27 ± 0.19
	P-value		0.793	0.527	0.025*	0.002*
P1NP (ng/mL)	Zoledronic Acid	88	27.44 ± 13.49	25.17 ± 9.85	24.71 ± 8.99	23.29 ± 7.88
	Denosumab	76	25.72 ± 12.58	23.73 ± 9.25	22.91 ± 8.36	21.69 ± 7.80
	P-value		0.402	0.339	0.187	0.194
NMID (ng/mL)	Zoledronic Acid	88	14.84 ± 5.65	13.33 ± 4.27	13.03 ± 3.67	12.48 ± 3.50
	Denosumab	76	16.17 ± 7.78	15.23 ± 8.26	13.98 ± 5.57	13.19 ± 5.30
	P-value		0.207	0.061	0.192	0.305
PTH (pg/mL)	Zoledronic Acid	88	43.34 ± 15.21	40.73 ± 12.46	37.52 ± 12.41	34.22 ± 9.53
	Denosumab	76	37.26 ± 10.17	34.18 ± 7.79	31.54 ± 6.34	28.94 ± 5.17
	P-value		0.004*	<0.001*	<0.001*	<0.001*

*P < 0.05 indicates statistical significance.

### 3.3 Bone mineral density

Lumbar spine BMD increased progressively in both groups post-treatment. No significant differences were observed between groups at 1 and 2 years post-treatment (P > 0.05). However, at 3 years post-treatment, lumbar spine BMD in the denosumab group was higher than that in the zoledronic acid group (0.85 ± 0.17 vs. 0.81 ± 0.14), although the difference did not reach statistical significance (P = 0.141). T-scores demonstrated a similar trend, with higher values observed in the denosumab group at year 3, though without achieving statistical significance.

**Figure 2:**
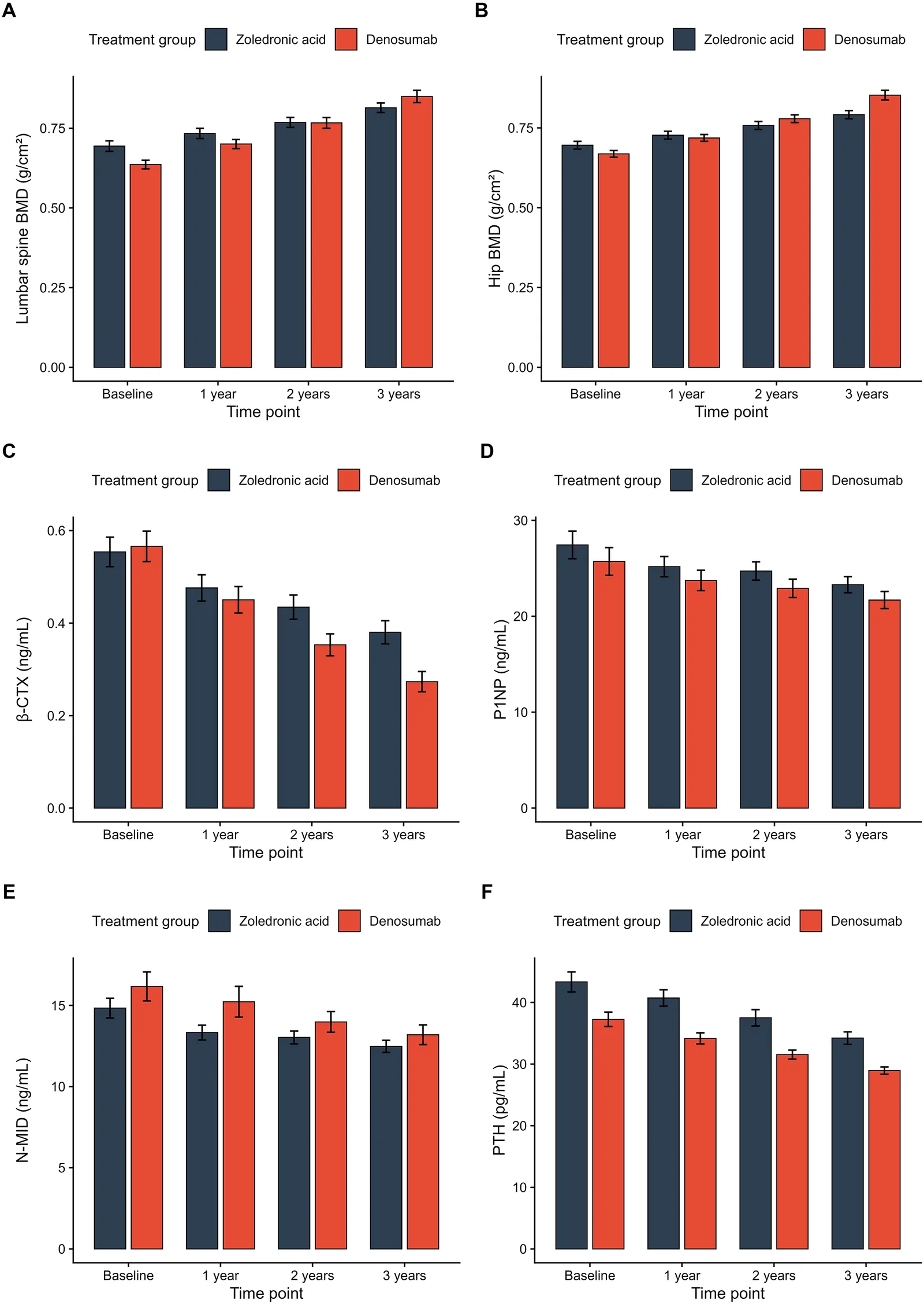
Longitudinal changes in bone mineral density and bone metabolism-related markers during the 3-year follow-up period. **(A)** Lumbar spine bone mineral density (BMD). **(B)** Hip BMD. **(C)** β-CTX. **(D)** P1NP. **(E)** N-MID. **(F)** PTH. Data are presented as mean ± standard error. BMD, bone mineral density; β-CTX, beta-C-terminal telopeptide of type I collagen; P1NP, procollagen type I N-terminal propeptide; PTH, parathyroid hormone.

Hip BMD also increased progressively in both groups post-treatment. No significant differences were observed between groups at 1 and 2 years post-treatment. However, at 3 years post-treatment, hip BMD in the denosumab group was significantly higher than that in the zoledronic acid group, with the difference reaching statistical significance (P = 0.002). After adjustment for age, sex, baseline PTH, lumbar spine BMD, lumbar spine T-score, and baseline hip BMD, treatment group remained significantly associated with hip BMD at 3 years (adjusted P < 0.001), **Table 2** and **Figure 2**.

**Table 2:** Comparison of bone mineral density before and after treatment.

Parameter	Treatment	n	Baseline	1 year	2 years	3 years
Lumbar BMD (g/cm²)	Zoledronic Acid	88	0.69 ± 0.15	0.73 ± 0.15	0.77 ± 0.15	0.81 ± 0.14
	Denosumab	76	0.64 ± 0.12	0.70 ± 0.12	0.77 ± 0.15	0.85 ± 0.17
	P-value		0.008*	0.130	0.951	0.141
Lumbar T-score	Zoledronic Acid	88	-2.88 ± 0.59	-2.71 ± 0.56	-2.58 ± 0.56	-2.40 ± 0.57
	Denosumab	76	-3.12 ± 0.59	-2.84 ± 0.60	-2.59 ± 0.65	-2.26 ± 0.68
	P-value		0.013*	0.140	0.900	0.158
Hip BMD (g/cm²)	Zoledronic Acid	88	0.70 ± 0.11	0.73 ± 0.12	0.76 ± 0.12	0.79 ± 0.12
	Denosumab	76	0.67 ± 0.09	0.72 ± 0.09	0.78 ± 0.10	0.85 ± 0.13
	P-value		0.101	0.603	0.229	0.002*
Hip T-score	Zoledronic Acid	88	-2.67 ± 0.56	-2.55 ± 0.55	-2.45 ± 0.58	-2.30 ± 0.59
	Denosumab	76	-2.74 ± 0.62	-2.52 ± 0.61	-2.29 ± 0.59	-2.02 ± 0.61
	P-value		0.432	0.757	0.094	0.003*

*P < 0.05 indicates statistical significance.

### 3.4 Safety profile

During the 3-year follow-up period, no cases of atypical femoral fracture, osteonecrosis of the jaw, or serious infections were reported in either treatment group. In the zoledronic acid group (n = 88), 32 patients experienced fever, 13 developed upper respiratory tract infections, and 17 reported generalized bone pain; no other significant adverse events were observed. In the denosumab group (n = 76), 8 patients experienced hip pain and 4 reported lower back pain; no other significant adverse events were noted. Overall, both treatments demonstrated favorable safety profiles.

## 4 Discussion

This retrospective study compared the long-term efficacy and safety of zoledronic acid and denosumab in the treatment of primary osteoporosis. These findings suggest that both medications affected bone turnover during follow-up. Notably, denosumab showed greater suppression of bone resorption, as reflected by significantly lower β-CTX levels at 2 and 3 years post-treatment, whereas no significant between-group differences were observed in P1NP levels.

Regarding bone turnover markers, this study found that denosumab produced a more potent suppression of β-CTX compared to zoledronic acid at 2 and 3 years post-treatment. This finding is closely related to the distinct mechanisms of action of the two medications. Denosumab is a fully humanized IgG2 monoclonal antibody that targets the receptor activator of nuclear factor-κB ligand (RANKL) expressed on osteoclast precursor cells (11, 12). RANKL is a critical signaling molecule in bone resorption. Upon binding to RANK receptors on osteoclast precursor cells, RANKL promotes osteoclast formation, differentiation, and activation. Denosumab mimics endogenous osteoprotegerin (OPG) by competitively binding to RANKL, thereby blocking the RANKL/RANK signaling pathway and inhibiting osteoclast activity at its origin. This specific targeted mechanism explains denosumab’s ability to reduce bone turnover markers and achieve sustained long-term increases in bone mineral density (22).

In contrast, zoledronic acid belongs to the nitrogen-containing bisphosphonate class (8, 9). During bone remodeling, it is absorbed by osteoclasts and internalized into the cells. Within the cell, zoledronic acid inhibits farnesyl diphosphate synthase (FPPS), thereby interfering with the mevalonate pathway (23). This pathway is essential for normal osteoclast function, and FPPS inhibition prevents the formation of necessary GTPase proteins, ultimately inducing osteoclast apoptosis. Although zoledronic acid effectively suppresses bone resorption, its mechanism of action involves affecting intracellular metabolic pathways within osteoclasts. Compared to denosumab’s direct blockade of signaling pathways, differences in potency and duration of action may exist. This is consistent with the observation in this study that denosumab demonstrated more potent suppression of β-CTX.

This study demonstrated that denosumab exhibits superior long-term efficacy over zoledronic acid in improving hip bone mineral density, consistent with findings from multiple previous studies. Kang et al. compared the efficacy of denosumab and zoledronic acid in postmenopausal osteoporosis patients and found that BMD was higher in the denosumab group after 2 years of treatment (24). A 2025 study by Ha et al. reached similar conclusions (25). The observation in this study that hip BMD in the denosumab group was significantly higher than in the zoledronic acid group at 3 years post-treatment aligns with these previous findings and further supports the superiority of denosumab as a potent anti-resorptive agent in terms of long-term efficacy.

Although previous studies have compared denosumab and zoledronic acid, the clinical context of these studies differs from that of the present cohort. Choi et al. mainly evaluated claims-based safety and fracture outcomes within one year after treatment initiation, while Son et al. focused on patients with acute osteoporotic vertebral compression fractures over a 12-month follow-up period (26). Kang et al. reported two-year data in Korean postmenopausal women. In contrast, the present study provides three-year longitudinal real-world data in Chinese patients with primary osteoporosis, integrating serial BMD, bone turnover markers, safety outcomes (27). Therefore, our findings should be interpreted as complementary evidence rather than as a replacement for previous studies.

This study demonstrated that neither treatment group experienced atypical femoral fractures, osteonecrosis of the jaw, or serious infections during the 3-year follow-up period, indicating favorable overall safety profiles. The incidence of adverse events with denosumab was not higher than that with zoledronic acid, consistent with previous reports (28, 29).

Several limitations of this study should be acknowledged. First, this was a single-center retrospective cohort study with a relatively limited sample size. Second, baseline differences in PTH, lumbar spine BMD, and lumbar spine T-score were observed between the two treatment groups, which may have introduced potential confounding; however, an additional ANCOVA was performed to adjust for these baseline differences. Third, systematic radiographic evaluation and fracture-related outcome data were not consistently available for all patients and therefore could not be comprehensively analyzed. Finally, some patients were excluded because of incomplete follow-up data, which is an inherent challenge in retrospective real-world studies. Future prospective multicenter studies with larger sample sizes, longer follow-up durations, standardized imaging assessment, and fracture-related endpoints are warranted to further validate these findings.

The findings of this study indicate that denosumab demonstrates superior long-term efficacy over zoledronic acid in suppressing bone turnover and improving hip bone mineral density. Both medications significantly increase lumbar spine and hip bone mineral density and exhibit excellent safety profiles. In clinical practice, denosumab may be considered a potent and rapidly acting anti-resorptive agent, particularly suitable for patients requiring rapid improvement in bone mineral density.

## Data Availability

The raw data supporting the conclusions of this article will be made available by the authors, without undue reservation.
